# Patient Engagement in Kidney Research: Opportunities and Challenges Ahead

**DOI:** 10.1177/2054358117740583

**Published:** 2017-11-29

**Authors:** Amber O. Molnar, Moumita Barua, Ana Konvalinka, Kara Schick-Makaroff

**Affiliations:** 1 Division of Nephrology, Department of Medicine, McMaster University, Hamilton, Ontario, Canada; 2 St Joseph’s Healthcare, Hamilton, Ontario, Canada; 3 Division of Nephrology, University Health Network, Toronto, Ontario, Canada; 4 Toronto General Hospital Research Institute, Toronto General Hospital, Ontario, Canada; 5 Department of Medicine, University of Toronto, Ontario, Canada; 6 Institute of Medical Sciences, University of Toronto, Ontario, Canada; 7 Faculty of Nursing, University of Alberta, Edmonton, Canada

**Keywords:** patient engagement, kidney, renal, research, translation, KRESCENT

## Abstract

**Purpose of Review:**

Patient engagement in research is increasingly recognized as an important component of the research process and may facilitate translation of research findings. To heighten awareness on this important topic, this review presents opportunities and challenges of patient engagement in research, drawing on specific examples from 4 areas of Canadian kidney research conducted by New Investigators in the Kidney Research Scientist Core Education and National Training (KRESCENT) Program.

**Sources of Information:**

Research expertise, published reports, peer-reviewed articles, and research funding body websites.

**Methods:**

In this review, the definition, purpose, and potential benefits of patient engagement in research are discussed. Approaches toward patient engagement that may help with translation and uptake of research findings into clinical practice are highlighted. Opportunities and challenges of patient engagement are presented in both basic science and clinical research with the following examples of kidney research: (1) precision care in focal and segmental glomerulosclerosis, (2) systems biology approaches to improve management of chronic kidney disease and enhance kidney graft survival, (3) reducing the incidence of suboptimal dialysis initiation, and (4) use of patient-reported outcome measures (PROMs) and patient-reported experience measures (PREMs) in kidney practice.

**Key Findings:**

Clinical research affords more obvious opportunities for patient engagement. The most obvious step at which to engage patients is in the setting of research priorities. Engagement at all stages of the research cycle may prove to be more challenging, and requires a detailed plan, along with funds and infrastructure to ensure that it is not merely tokenistic. Basic science research is several steps removed from the clinical application and involves complex scientific concepts, which makes patient engagement inherently more difficult.

**Limitations:**

This is a narrative review of the literature that has been partly influenced by the perspectives and experiences of the authors and focuses on research conducted by the authors. The evidence base to support the suggested benefits of patient engagement in research is currently limited.

**Implications:**

The formal incorporation of patients’ priorities, perspectives, and experiences is now recognized as a key component of the research process. If patients and researchers are able to effectively work together, this could enhance research quality and efficiency. To effectively engage patients, proper infrastructure and dedicated funding are needed. Going forward, a rigorous evaluation of patient engagement strategies and their effectiveness will be needed.

## Abrégé

**Objet de la revue:**

La participation des patients au processus de recherche tend à se généraliser; elle peut en effet faciliter l’application des résultats à la pratique clinique. Afin de sensibiliser la communauté scientifique à ce sujet, notre revue présente diverses occasions d’intégrer les patients au processus de recherche, de même que les défis posés par cette intégration. Nos constats sont tirés d’exemples issus de quatre domaines de recherche en néphropathologie conduite au Canada par les nouveaux chercheurs du programme KRESCENT (Kidney Research Scientist Core Education and National Training).

**Sources:**

Nous avons consulté des chercheurs, des rapports, des articles évalués par les pairs et des sites Web d’organisations finançant la recherche.

**Méthodologie:**

Sont discutés dans la présente revue la définition, l’objectif et les bienfaits potentiels de la participation de patients à la recherche. On a mis l’accent sur certaines stratégies susceptibles de favoriser la transposition et l’application des résultats de recherche dans la pratique clinique. Les occasions et défis inhérents à cette pratique sont présentés tant en recherche fondamentale qu’en recherche clinique, et ce, dans quatre domaines de la recherche en néphropathologie : 1) les soins de précision en hyalinose segmentaire et focale, 2) les approches de la biologie systémique qui améliorent la gestion des maladies rénales chroniques et les chances de survie du greffon, 3) la diminution de l’incidence des amorces de dialyse suboptimales, et 4) la prise en compte des résultats et expériences déclarés par les patients (MRDP et MEDP) dans la pratique en néphrologie.

**Principaux constats:**

La recherche clinique offre les occasions les plus concevables d’intégrer les patients au processus. L’étape de l’établissement des priorités de recherche est la plus opportune à l’incorporation du patient au projet. L’intégration des patients à un moment ou à un autre du cycle de recherche peut se révéler difficile; elle requiert un plan détaillé, des infrastructures adéquates et des ressources financières convenables pour que sa portée dépasse la simple symbolique. La recherche fondamentale se trouve quant à elle plus éloignée de l’application clinique; et comme elle repose sur des concepts scientifiques complexes, l’incorporation des patients y est plus épineuse.

**Limites:**

Il s’agit d’une revue narrative de la littérature en partie influencée par les recherches menées par les auteurs, leurs expériences et leurs points de vue. Qui plus est, les données probantes confirmant les bienfaits plausibles de la participation des patients à la recherche sont à ce jour limitées.

**Implications:**

L’incorporation formelle des priorités, du point de vue et du vécu des patients au processus de recherche se voit désormais considérée comme un élément clé de celui-ci. Une collaboration resserrée entre patients et chercheurs ne pourra qu’accroître la qualité et l’efficacité de la recherche. Par contre, l’intégration opérante des patients au processus de recherche requiert infrastructures et financement appropriés. À l’avenir, il faudra évaluer rigoureusement des stratégies d’intégration des patients au processus de recherche et en mesurer l’efficacité.

## What was known before

There is increasing awareness of the importance of patient engagement in research. Funding bodies have recognized this and have allocated large sums of money to patient-oriented research. Patient engagement is thought to serve as an important facilitator of knowledge translation.

## What this adds

Through specific examples of Canadian nephrology research programs, opportunities and challenges for patient engagement in research are presented, with a particular emphasis on how patient engagement may help to bridge the “death valleys” of research, and how patient engagement differs in basic science and clinic research.

## Introduction

Over the past decade, there has been a movement toward increased patient engagement in research. Patient engagement has been defined as patients having a “meaningful and active collaboration in governance, priority setting, conducting research and knowledge translation.”^1,2^ Guiding principles that underpin the integration of patient engagement in research are outlined in Table 1. The rise of patient engagement in research coincides with the movement toward person-centered care, which places an emphasis on clinical care that is tailored to and respectful of an individual’s preferences and values.^3,4^ Researchers and national funding agencies alike have become increasingly aware of the limitations and failures of the traditional research model that is solely driven by the researcher. Collectively, patient-oriented research (POR) addresses these limitations in a manner that “engages patients as partners, focuses on patient-identified priorities, and improves patient outcomes.”^1^

**Table 1.** Guiding Principles for Integrating Patient Engagement in Research.

Principle	Definition
Inclusiveness	Patient engagement in research integrates a diversity of patient perspectives and research is reflective of their contributions; that is, patients are bringing their lives into this.
Support	Adequate support and flexibility are provided to patient participants to ensure that they can contribute fully to discussions and decisions. This implies creating safe environments that promote honest interactions, cultural competence, training, and education. Support also implies financial compensation for their involvement.
Mutual respect	Researchers, practitioners and patients acknowledge and value each other’s expertise and experiential knowledge.
Co-build	Patients, researchers, and practitioners work together from the beginning to identify problems and gaps, set priorities for research, and work together to produce and implement solutions.

*Source.* Adapted from Canada’s Strategy for Patient-Oriented Research: Patient Engagement Framework.^1^

Patients, through their experiential knowledge, provide a unique and essential perspective. It is increasingly recognized that researchers often fail to identify research questions that are of greatest importance to patients. Prior and current randomized trials in dialysis patients tend to focus on outcomes of surrogate biochemical measurements and mortality. However, a recent survey of dialysis patients and their caregivers found that the top 10 outcomes ranked by patients were primarily relevant to daily symptom burden and well-being. Surprisingly, the outcome of mortality was only ranked 14th.^5^ Researchers may also fail to recognize the needs and challenges of patients, which may ultimately impact study execution. For example, patients with advanced kidney disease often have multiple comorbidities along with a high symptom burden.^6-8^ As well, studies report that patients with lower socioeconomic status have more severe kidney disease on presentation to nephrology clinics, and patients with lower educational attainment are more likely to have kidney disease.^9,10^ This may hinder enrollment and retention of patients in research studies for a number of reasons (ie, failure to understand research information materials and consent forms, or an inability to attend study visits). A number of studies in the dialysis patient population have faced challenges with lower than anticipated recruitment and high attrition, ultimately resulting in failure of the study to definitively answer the research question.^11-14^ Engagement of patients in the planning and execution of kidney research may not only lead to a research plan that is responsive to patients’ concerns but also effectively increase participation and uptake. Patients are the ultimate recipients of evidence-informed practice; therefore, ensuring that research aligns with patients’ needs may strengthen knowledge translation along the research to practice continuum.

There are 2 recognized critical time points at which research commonly fails to progress along the continuum. These 2 time points are known as the “Death Valleys” of research. Valley 1 represents the challenge of translating basic biomedical discoveries made in the laboratory to the clinical realm. Valley 2 represents the challenge to synthesize, disseminate, and integrate clinical research results into clinical practice, health care decision making, and policy.^15^ In 2014, it was reported that approximately 85% of dollars invested into biomedical research is wasted, amounting to $200 billion worldwide.^16^ A series published in the Lancet identified wasteful practices that occur all along the scientific process, labeling waste as resources that are used in an unjustifiable and avoidable manner. The authors appropriately recognize that not all biomedical research leads to exciting, positive results nor will all research have a direct clinical application, and this research is not necessarily considered wasteful as long as it is legitimate. A complex interplay of economic, social, cultural, and political factors was identified as the cause of suboptimal funding, conduct, and regulation of the research process. Examples of current processes potentially leading to waste are nontransparent ranking of research priorities by funding bodies, a lack of information sharing on research that is in progress potentially leading to duplication, and flawed methodologies due to improper training, convenience, or pressures to publish.^17,18^ Patient engagement offers one opportunity to help address these systemic issues and reduce waste.

Kish^19^ argues that “if patient engagement were a drug, it would be the blockbuster drug of the century and malpractice not to use it.” Internationally, research funding agencies have created strategies for patient engagement and POR in an effort to improve uptake of research findings into patient care and to align the research process with the concept of person-centered care. In the United Kingdom, INVOLVE is an agency publicly funded by the National Institute for Health Research to support active public involvement in research.^20^ In the United States, the Patient-Centered Outcomes Research Institute (PCORI) supports research guided by patients, caregivers, clinicians, and other health care stakeholders.^21^ In Canada, the Canadian Institutes of Health Research (CIHR) has launched a Strategy for Patient-Oriented Research (SPOR), with dedicated funding of SPOR networks in chronic disease. A SPOR network dedicated to kidney disease (Canadians Seeking Solutions and Innovations to Overcome Chronic Kidney Disease [Can-SOLVE CKD]) was recently funded approximately 40 million dollars plus matched funds. Can-SOLVE CKD is an innovative partnership of patients, researchers, practitioners, policy makers, industry, and renal agencies collaboratively focused on transforming the care of people affected by kidney disease in Canada.^22^

Taken together, active patient engagement in research consists of a mutually beneficial relationship between researchers and patients. Researchers benefit if the likelihood of producing meaningful and impactful research is increased. In turn, patients benefit if research dollars and resources are focused on research that addresses patients’ needs and ultimately improves patient care. The potential benefits of patient engagement in research are increased patient enrollment, decreased attrition, improved dissemination of findings, and research endeavors that are responsive to patients’ priorities.^23^ There is also the consideration that patient engagement is now deemed favorable, if not, mandated by funding agencies.

While patient engagement in research is not a new concept, the optimum strategies to engage patients in kidney research have not been fully explored.^23^ In this review, 4 areas of Canadian kidney research conducted by New Investigators in the Kidney Research Scientist Core Education and National Training (KRESCENT) Program are highlighted. The research presented spans the continuum from basic science to clinical research involving patient-reported outcome measures. With each area of research, opportunities and challenges for patient engagement are discussed. Furthermore, strategies of engaging patients to assist with knowledge translation and overcoming the so-called “Death Valleys” of research are discussed.

### Helping to Bridge Death Valley 1: Patient Engagement in Basic and Translational Kidney Research

Patient and public engagement is lagging in laboratory-based research. The importance of basic research is easy for the lay public to recognize when considering major medical discoveries stemming from basic science work, including antimicrobial therapies, vaccinations, insulin, and the genetic code. However, patient engagement in basic research may prove to be challenging given the scientific complexity of the research questions and the methodologies to address them. Basic science research is also often the foundation on which scientific discovery is made and therefore an early stage at which to influence research priorities. Herein, opportunities to incorporate patient perspectives in basic and translational renal research are highlighted by presenting the work of 2 Canadian clinician scientists (M.B. and A.K.).

#### Precision care in focal and segmental glomerulosclerosis

Genetics is the starting point that governs and coordinates the biological processes of an organism. The impetus for sequencing the first human genome was driven by stakeholder engagement—the US Department of Energy wanted to understand how to protect the genome from the mutagenizing effects of radiation and entered into a memorandum of understanding with the National Institutes of Health (NIH).^24^ Decades and hundreds of millions of dollars of investment later, whole human genomes are now routinely being sequenced by research laboratories.^25,26^ By sequencing the human genome, the code to an individual is being unraveled to correlate it with health and disease.

Precision care and patient-centered care are terms that have recently reached ubiquity among different groups of medical researchers and are interrelated concepts. Patients need more concrete data regarding disease cause, course, and self-identified important treatment adverse events for effective shared and informed decision making. The development of genetic testing in focal and segmental glomerulosclerosis (FSGS) as a noninvasive diagnostic and prognostic test is important to spare individuals from toxic, ineffective therapies; family planning; and importantly, to identify suitable at-risk relatives for kidney donation if and when end-stage renal disease (ESRD) occurs. The generation of more scientific evidence to guide clinical interpretation would facilitate physician and government buy in for this screening to occur.

A KRESCENT New Investigator (M.B.)–led laboratory is using powerful methods of genetic analyses to understand the causes and molecular basis of FSGS. Within this research program, it is hypothesized that gene mutations alone or in aggregate play an important and significant role in FSGS causation and susceptibility. The major objectives are to (1) develop whole genome sequencing (WGS) as a noninvasive diagnostic and prognostic tool, (2) integrate genetic data with detailed clinical outcomes, (3) identify clinician and patient-centered barriers to the inclusion of genetic testing as a biomarker, and (4) understand disease mechanisms.

The Glomerulonephritis (GN) Research Program at University Health Network (UHN) in Toronto was first founded by Dr Daniel Cattran in the 1970s to better understand the natural history and response to treatment of different GN disorders. In conjunction with the Hereditary Kidney Disease Program, founded by Dr York Pei, DNA samples belonging to study participants have been collected since the early 2000s. These 2 priority programs within the Division of Nephrology at UHN have served as a powerful resource for numerous translational and clinical studies in GN. M.B.’s laboratory has performed whole exome and genome sequencing in this well-phenotyped cohort and found a genetic cause in a significant proportion of familial and sporadic cases.^27^ Importantly, the laboratory has begun to derive genotype-phenotype associations, which will help guide evidence-based treatment practices.

Alongside the potential for WGS to diagnose and optimize care for individuals with FSGS, however, a number of complex features of this technology may alter its acceptability from a patient and clinician perspective. Key among these challenges are (1) the identification of complex variants (ie, predictive secondary variants and variants of uncertain clinical significance), (2) the emerging data sharing imperative, and (3) the multistep process of care and multidisciplinary expertise that is required to support WGS implementation. To date, these complexities are not well understood. The laboratory of M.B. is beginning to collaborate with specialized social scientists to explore providers’ and patients’ preferences regarding educational content and strategies, preferences for actual receipt of complex variants, perspectives on data sharing with centralized repositories, and perspectives on the multistep clinical process of care required for successful implementation.

Long term, the identification of genetic causes serves as the starting point to understand pathobiology in clinically relevant genetic models.^27-31^ This work spans the continuum of clinical to translational research.^32^ Genetic studies in FSGS have already revealed significant insight into glomerular pathobiology. FSGS has long been viewed as a podocyte disorder because of its ultrastructural characteristics and numerous genetic studies implicating mutations in podocyte proteins in disease.^33-38^ More recently, the work of M.B.’s laboratory and others have shown that mutations in genes expressed in tubules and during kidney development are also associated with the FSGS lesion.^28,39^ As a result of this finding, future work will explore the abnormal processes in development and repair that lead to the late-onset FSGS lesion in cell and animal models. Ultimately, better understanding of disease mechanism will facilitate the development of therapeutics, which is lacking and desperately needed in this patient population.

#### Systems biology approaches to improve management of CKD and enhance kidney graft survival

The research program led by KRESCENT New Investigator, A.K., applies advanced proteomics analysis and systems biology to the study of kidney disease to improve diagnosis, inform treatment, and assess prognosis. The research program strives to bridge the gap between basic discoveries and the clinic.

The research program has 3 major themes that address unmet clinical needs in CKD and kidney transplantation. The first theme focuses on the lack of markers of kidney angiotensin II (AngII) activity that could guide therapy with renin-angiotensin system inhibitors and identify progression of kidney fibrosis. To date, this research team has defined proteins significantly regulated by AngII in primary kidney cells^40^ and demonstrated that these “AngII signature proteins” reflected kidney fibrosis in animal models of CKD.^40,41^ These proteins may also reflect kidney fibrosis in patients with CKD^42^ and kidney transplant. To bridge the gap between AngII signature proteins and their clinical applications, the lab is exploring their use with antifibrotic treatments and turning to patients’ perspectives on research priorities, through involvement with the Canadian National Transplant Research Program.^43^

The key unmet need in kidney transplantation is the improved monitoring and treatment of antibody-mediated rejection (ABMR). ABMR is caused by antibodies against the human leukocyte antigens (HLAs) and non-HLA antigens expressed on the graft endothelium.^44-48^ Improved understanding of pathogenic HLA and non-HLA antibodies and development of effective treatments could prolong allograft survival.^49^ Improving allograft survival has been identified as a top priority by patients with a kidney transplant.^50^ The next step is to study the interaction between circulating HLA and non-HLA antibodies and graft antigens to define clinically useful assays for monitoring ABMR and to develop novel therapeutics. This work may directly impact patients and reflect their priorities.

The final theme of the research program involves the investigation of early mechanisms of diabetic kidney injury. Naturally occurring peptides in the urine of healthy controls and patients with early diabetes mellitus type I can pinpoint proteases active in the kidney. These proteases may uncover the earliest effects of hyperglycemia on the kidney and the potential treatments to prevent diabetic nephropathy.^51^ This is a collaborative project with the Adolescent Diabetes Cardio-Renal Intervention Trial, under the umbrella of the SPOR network Can-SOLVE CKD, which is informed by patients’ priorities.

*What are the challenges for enacting patient engagement in translation of basic research?* The large gap between basic science and the clinical realm was recognized a decade ago.^52^ Basic scientific projects were perceived to have little relevance to patient care, and scientists were largely unaware of the unmet clinical needs or the patients’ priorities. The animal models and immortalized cells fail to recapitulate biology relevant to patients and tend to be dissociated from patient care.^53^ The engagement of patients in all phases of scientific research has been proposed; however, patient engagement during data collection and analysis is challenging. The counterpoint to pursuing translational research is the need to solve fundamental scientific questions, which may not directly impact patients. Intriguingly, these endeavors have produced some of the most transformative tools that have influenced the health of humans (eg, the discovery of CRISPR-Cas9 and DNA).^54,55^

*How can we prevent the loss of information in translation?* The field of translational research has heralded a new breed of researcher, the clinician scientist. These are health care providers who are trained in science and intended to bridge the gap between “bench-and-bedside” by understanding the unmet clinical needs of their patients and knowing how to address them in the lab. Multidisciplinary teams that include patients and caregivers, health care providers, scientists, biostatisticians, and so on, may facilitate translational research. Basic scientific discoveries can progress across Valley 1 only with the integration of multiple disciplines and stakeholders. Our research programs have been embedded into similar multidisciplinary networks.

Among the members of such multidisciplinary teams, patients are increasingly recognized as important stakeholders and partners in translational research, who are willing and able to direct research priorities.^56^ This is evident from large funding bodies in Canada, the United States, and the United Kingdom, who support active public involvement in research. Patient engagement in research can take the form of consultation, partnership, or patient-led research.^57^ Although patients have been engaged in setting research priorities,^58-61^ the most effective way to engage patients in basic biomedical research is largely unexplored.

Much of the work led by A.K. is performed using patients’ biospecimens. The ability to analyze patients’ biospecimens matched to clinical information may overcome some limitations of animal models and transformed cells.^53^ For example, studying molecular signatures in patient-derived biospecimens is an attractive gateway into precision medicine.^62,63^ In the context of patient engagement, patients can provide specimens for research, but they can also be actively involved in determining the best use of those specimens, educating others on the importance of providing specimens, and helping to disseminate findings of the research conducted.

### Helping to Bridge Death Valley 2: Patient Engagement in Clinical Kidney Research

Compared with basic or translational research, there are more obvious opportunities for patient engagement in kidney clinical research. Active participation of kidney patients in the ranking of clinical research priorities and the design of clinical research studies are the 2 most obvious areas for patient engagement. The role of patients in the execution phase of studies, that is, helping with patient enrollment, or how patients may help with broader dissemination of study findings, is an evolving field in public and patient engagement.^64,65^ Two examples of Canadian kidney researchers (A.O.M. and K.S.-M.) who are working to incorporate patient engagement into their clinical research programs are presented.

#### Reducing the incidence of suboptimal dialysis initiation

In 2013 alone, more than 5300 patients in Canada initiated some form of renal replacement therapy (RRT; dialysis or preemptive kidney transplant).^66^ Unfortunately, many of these patients initiated RRT suboptimally, commonly defined as the initiation of dialysis during a hospitalization or with an unplanned central venous catheter (CVC).^67-70^ Late referral to a nephrologist is associated with a higher likelihood of starting RRT suboptimally.^67,69,71-73^ However, patients referred early are still more likely to start RRT suboptimally rather than optimally. Based on a multicenter Canadian study from 2006, 60.5% of dialysis starts are suboptimal, and more than 50% of patients starting dialysis suboptimally are known to a nephrologist for over a year.^68,69^ Suboptimal dialysis starts are of concern because they are expensive,^19,70,74^ and are associated with increased patient morbidity and mortality.^69,70,75-78^ Given this, reducing the incidence of suboptimal dialysis starts is of importance to multiple stakeholders (ie, clinicians, funders of renal and dialysis care, and patients). How may a research program be implemented that will best address this issue and incorporate patient engagement?

To reduce the incidence of suboptimal dialysis starts, the question of why they occur so frequently must first be addressed. A KRESCENT New Investigator (A.O.M.)–led research program proposes to answer this question by performing a prospective cohort study examining risk factors for suboptimal dialysis initiation, with a focus on *modifiable and actionable* risk factors. Potential risk factors include not only demographics and comorbidities but also factors that focus on preventive care, health literacy, adherence with recommended treatments, and patient-provider communication. Risk factors of interest highlight communication, self-management, and informed decision making based on prior work by others that employed patient engagement to define the top 10 research uncertainties for patients on or nearing dialysis.^58^ One of the top uncertainties identified by patients, caregivers, and health care providers was how to best enhance communication between patients and health care providers to maximize patient participation with regard to RRT decision making.

Another important factor when considering the high incidence of suboptimal dialysis starts is that physicians and renal networks have largely constructed and defined this outcome. Health care providers assume that the avoidance of a suboptimal dialysis start, as currently defined, is of utmost importance to patients given the association with increased mortality, morbidity and reduced quality of life;^69,70,75-78^ however, patients were not engaged when this outcome was defined. Therefore, patients may not actually recognize this to be an important outcome, which could be a contributor in and of itself to the high incidence of suboptimal dialysis starts. Patients may have other priorities and values that health care professionals have failed to recognize, or simply may not consider this outcome important due to a lack of education. Whether patients understand that suboptimal dialysis starts are potentially detrimental to their health, and whether this outcome, as currently defined, is considered important to patients, will be determined by surveying patients.

Once the risk factors of greatest importance have been identified, the next step in the research plan will be to design and test a multifaceted intervention with the aim of reducing the incidence of suboptimal dialysis starts. A group of patients with advanced kidney disease, along with nephrologists, renal nurses, and a representative from the provincial renal network, will be asked to provide input regarding the design of the intervention and the research protocol. Engagement from multiple stakeholders is particularly important because the intervention will likely primarily address patient-provider communication and patient education, which require time and active participation on the part of both clinicians and patients.

Patient engagement will hopefully enhance the success of the research plan by increasing study enrollment, and enhancing the likelihood of designing an effective intervention that will be successfully translated into clinical practice (bridging Death Valley 2). Still, effective patient engagement will not be without its challenges. Seeking input from multiple stakeholders will require added investment of time and resources. However, the investment should prove worthwhile if patient engagement helps produce an effective intervention that meets the needs of patients, enhances patient self-efficacy and shared decision making, and ultimately achieves the end goal of reducing the incidence of suboptimal dialysis starts.

#### Use of PROMs and PREMs in kidney practice

Internationally, the Institute for Healthcare Improvement urges health organizations and researchers to focus on what matters to patients. It has been shown that patients report better outcomes and experiences of care when clinicians are curious about and act on patients’ health priorities and concerns.^79^ Nationally, the Canadian Institute for Health Information (CIHI) highlights patient-reported outcome measures (PROMs) and patient-reported experience measures (PREMs) as *essential* to understanding whether health services make a difference to patients’ health and quality of life.^80,81^ PROMs refer to “self-report instruments used to obtain health care recipients’ appraisals of health outcomes relevant to their quality of life” and PREMs are “self-report instruments used to obtain patients’ appraisals of their experience and satisfaction with the quality of care and services.”^82^ Taken together, PROMs and PREMs offer reports from kidney patients which help support shared decision making in health service research and clinical practice.

To date, uptake of patient engagement in kidney research has been slow.^83^ Use of PROMs and PREMs, both at individual and aggregate levels, in kidney research, practice, and health care administration offer *significant opportunities* to place patients’ own reports of their health outcomes and experiences at the core of nephrology health service delivery. However, in spite of mounting support for PROMs and PREMs to enhance person-centered care,^58,83-85^ collection and reporting of PROs is *not* routinely integrated into kidney care across in Canada. Accordingly, optimal strategies to integrate PROs into care remain unknown.^86,87^

Guided by an integrated knowledge translation (iKT) strategy,^88^ interdisciplinary teams led by a KRESCENT New Investigator (K.S.-M.) are currently addressing this gap in our understanding by (1) conducting a realist synthesis of empirical research on effective strategies that guide how PROMs and PREMs may be fully utilized in kidney settings (funded by Kidney Foundation of Canada^89^) and (2) exploring *how* integration of electronically provided patient-reported outcomes (ePROs) may provide the best care and health possible to kidney patients on home dialysis modalities (funded by CIHR). These research projects are being undertaken collaboratively with Patient Advisory Committees, clinician knowledge users, CIHI, PROM and PREM experts, government administrators, and technology industry partners.

One of the barriers to uptake of PROMs and PREMs is the concern of patient burden. However, this worry is unsubstantiated and previous research with kidney patients has shown the opposite: People living with kidney disease are often not only willing but want their perspectives to be incorporated into their care.^90,91^ Another barrier to integration of PROMs and PREMs in kidney research is the persistent judgment of findings as biased.^92^ This critique highlights that indeed bias is inherent in personal reports by those living with kidney disease. However, principles of patient engagement acknowledge the importance of firsthand reports of health status, and health outcomes or experiences, as important elements frequently overlooked. PROMs and PREMs present opportunities to facilitate patient engagement and support person-centered care in kidney health service research and clinical practice.

## Conclusions

In this review, patient engagement in research has been highlighted within the context of 4 Canadian kidney research programs led by KRESCENT New Investigators. The overarching opportunities and challenges of patient engagement across the “Death Valleys” are summarized in Table 2. The formal incorporation of patients’ priorities, perspectives, and experiences is now recognized as a key component of the research process.

**Table 2.** Patient Engagement to Bridge Death Valleys in Kidney Research.

Death Valleys	Opportunities	Challenges
Death Valley 1: Basic biomedical research to clinical science and knowledge	Stakeholder engagement can drive innovation (ie, sequencing of first human genome) Kidney patients’ main concerns can provide direction for future research priorities Unmet kidney care needs can be informed with community/patient consultation and addressed in early phases of scientific exploration Biochemical and molecular approaches to answer clinical questions may inform personalized monitoring and treatment of patients Stakeholder partnerships can be very broad encompassing patients, caregivers, physicians, basic and clinical scientists, biostatisticians and computational biologists, industry, engineers, and so on.	Unchartered exploration of patient engagement throughout the research cycle Research is several steps removed from clinical application Transparency with patients about the delay from bench to bedside is imperative It is challenging to engage patient stakeholders during design, execution, and analysis of a study, and perhaps easier to envision engagement in dissemination and implementation Patient partners may be intimidated by the abstract science Projects aimed at fundamental scientific questions may not have any direct relevance to patient care or unmet clinical needs
Death Valley 2: Clinical science and knowledge to health decision making and clinical practice	Participation of kidney patients in the ranking of clinical research priorities Provides kidney patients and other stakeholders with knowledge and influence, gives researchers greater insight into their area of study, and may meet funding body requirements Patient engagement can be integrated throughout the research cycle Enhancement of research uptake with endorsement by patients who will ultimately benefit from findings PROMs and PREMs facilitate kidney patient engagement and support person-centered care in health service research and clinical practice	Token representation of patient engagement Pervasive myths of patient burden Additional time, finances, and resources are required for full integration of patient engagement spanning from design to implementation Education of all team members of the role of patients in the team Kidney researchers require training, infrastructure, and resources to effectively engage patients in research endeavors

*Note.* PROMs = patient-reported outcome measures; PREMs = patient-reported experience measures.

The rise of patient engagement in research is underscored by national research funding agencies dedicating huge sums of money specifically toward POR. As an example, Canada’s primary national research funding body, CIHR, recently funded a large Canadian POR research network committed to improving outcomes in patients with kidney disease (Can-SOLVE CKD). Dedicated funding networks, such as CAN-SOLVE CKD, should help to overcome some of the barriers to patient engagement, such as the need for infrastructure and resources to effectively engage patients.

Clinical research, with its immediate linkage to patients, is “heading the charge” of patient engagement. Basic science research may afford less obvious and more limited opportunities for patient engagement, but patient perspectives can still be incorporated. A number of complex features may be associated with developing diagnostic tools and therapeutic agents in the laboratory that alter its acceptability from both a patient and clinician perspective. Genomics research, for instance, raises the issue of complex genetic variants, including predictive secondary variants (eg, incidental finding of a high-risk BRCA1 variant) and variants of uncertain clinical significance. Patient and end-user engagement is required to best utilize genetic information and anticipate and deal with potentially unintended consequences.

Currently, there are many hypothesized benefits of patient engagement in research, but its true value has yet to be confirmed. As the field of patient-oriented kidney research advances, rigorous research is needed not only to determine optimal methods and approaches of patient engagement but also to evaluate outcomes associated with patient engagement. To date, this has largely been unexplored in both patient engagement work and kidney patient engaged research. Evaluation frameworks should include clear goals for patient engagement in research, and incorporate subjective measures, such as patient participant satisfaction, and objective measures, such as patient recruitment/study retention. Esmail et al^93^ recommend that for such evaluation to move forward, 4 key elements are essential. First, an evaluative framework for patient engagement in research should be selected prior to engaging in research activities. Second, validated tools should be used in this evaluation. Third, evaluations should be conducted at regularly scheduled times during the process of patient engagement. And fourth, the context and processes of engagement must be documented as an essential component of evaluation. These suggestions for evaluation could be used to help strengthen the evidence base for the practice of patient engagement in research.

Moving forward, patient engagement should be considered an essential component of the development and execution of meaningful research programs. It should be viewed as a symbiotic relationship between researchers and patients that may help bridge the so-called “Death Valleys” of research. As a research community, we need to embrace patient engagement and work toward developing optimal, impactful, and efficient strategies. Funding, infrastructure, and the integration of evaluative frameworks will all help to refine and optimize patient engagement strategies and overcome barriers.
